# Evaluating the performance of Affymetrix SNP Array 6.0 platform with 400 Japanese individuals

**DOI:** 10.1186/1471-2164-9-431

**Published:** 2008-09-22

**Authors:** Nao Nishida, Asako Koike, Atsushi Tajima, Yuko Ogasawara, Yoshimi Ishibashi, Yasuka Uehara, Ituro Inoue, Katsushi Tokunaga

**Affiliations:** 1 Department of Human Genetics, Graduate School of Medicine, The University of Tokyo, Tokyo, Japan; 2 Central Research Laboratory, Hitachi Ltd, Tokyo, Japan; 3 Division of Molecular Life Science, School of Medicine, Tokai University, Isehara, Japan

## Abstract

**Background:**

With improvements in genotyping technologies, genome-wide association studies with hundreds of thousands of SNPs allow the identification of candidate genetic loci for multifactorial diseases in different populations. However, genotyping errors caused by genotyping platforms or genotype calling algorithms may lead to inflation of false associations between markers and phenotypes. In addition, the number of SNPs available for genome-wide association studies in the Japanese population has been investigated using only 45 samples in the HapMap project, which could lead to an inaccurate estimation of the number of SNPs with low minor allele frequencies. We genotyped 400 Japanese samples in order to estimate the number of SNPs available for genome-wide association studies in the Japanese population and to examine the performance of the current SNP Array 6.0 platform and the genotype calling algorithm "Birdseed".

**Results:**

About 20% of the 909,622 SNP markers on the array were revealed to be monomorphic in the Japanese population. Consequently, 661,599 SNPs were available for genome-wide association studies in the Japanese population, after excluding the poorly behaving SNPs. The Birdseed algorithm accurately determined the genotype calls of each sample with a high overall call rate of over 99.5% and a high concordance rate of over 99.8% using more than 48 samples after removing low-quality samples by adjusting QC criteria.

**Conclusion:**

Our results confirmed that the SNP Array 6.0 platform reached the level reported by the manufacturer, and thus genome-wide association studies using the SNP Array 6.0 platform have considerable potential to identify candidate susceptibility or resistance genetic factors for multifactorial diseases in the Japanese population, as well as in other populations.

## Background

Together with technology developments on large-scale single nucleotide polymorphism (SNP) genotyping [1,2], there have been a number of genome-wide association studies (GWAS) to identify candidate susceptibility or resistance genetic factors for multifactorial diseases [3-7]. It is estimated that eleven million SNPs with a greater than 1% minor allele frequency (MAF) are located in the human genome [8]. Over six million SNPs have been uploaded on public SNP databases through the Human Genome Project and international SNP discovery projects. Among these SNPs, over 900 K SNPs across the human genome are selected with an average MAF of 19.6%, 18.2% and 20.6% in the HapMap Caucasians, Asians and Africans, respectively, and can be simultaneously genotyped using Affymetrix Genome-Wide Human SNP Array 6.0 platform [9]. Several studies have evaluated the coverage of commercial platforms using HapMap population data and genotype data of non-reference Caucasian populations [10-12]. Results from these studies indicated that in a non-reference Caucasian population, as well as the HapMap populations, commercial SNP typing platforms offered similar levels of genome coverage. However, the number of genotyped Japanese individuals in the HapMap project was only 45 samples, which may lead to inaccurate estimation of the number of SNPs with low MAF in the Japanese population.

The SNP Array 6.0 platform offers the genotype calling algorithm "Birdseed" to determine the genotypes of 909,622 SNPs [9]. The Birdseed algorithm performs a multiple-chip analysis to estimate signal intensity for each allele of each SNP, fitting probe-specific effects to increase precision, and then makes genotype calls by fitting a Gaussian mixture model in the two-dimensional A-signal vs. B-signal space, using SNP-specific models to improve accuracy. There was a report that 45% of SNPs observed to be significantly associated with the disease did not agree with Hardy-Weinberg equilibrium (HWE) using the previous version of Mapping 500 K Array set [13]. Some of the miss-called SNPs would be induced by genotype calling algorithms and are likely to be ranked as significantly associated with the disease (false-positive). Therefore, there are strong demands for accurate genotype calls using the Birdseed algorithm.

The SNP Array 6.0 platform has three check points prior to hybridization on GeneChip arrays in order to exclude experimental errors; PCR amplicon size check by electropherograms, DNase I digested fragment size check by electropherograms and quantity check of the purified PCR products. The platform also includes Quality Control (QC) probes for 3,022 SNPs to assess the overall quality for a sample based on the Dynamic Model (DM) algorithm. There are assay criteria to exclude experimental errors and low-quality samples; however, we empirically know that some samples, which pass these criteria, have low-quality genotyping results.

In this study, we genotyped 400 non-HapMap Japanese samples using the SNP Array 6.0 platform in order to evaluate the number of SNPs available for GWAS in the Japanese population, to examine an appropriate approach for acquiring accurate genotype calls using the Birdseed genotype calling algorithm, and to evaluate the assay criteria for preventing low-quality genotyping data.

## Results

### Genotyping 400 Japanese samples using SNP Array 6.0 platform

We collected 2 sets of 200 Japanese samples for genotyping using the SNP Array 6.0 platform. The average concentration of genomic DNA for the 1^st ^set of 200 samples was 54.8 ng/μl and that for the 2^nd ^set of 200 samples was 52.7 ng/μl. One of the critical points for the SNP Array 6.0 platform to acquire high quality genotyping data is to prepare a uniform quantity of 250 ng genomic DNA for Nsp ^I ^and Sty ^I ^digestion steps. When an almost 10-fold excess amount of genomic DNA was used, the average overall call rate drastically decreased to about 80% for both Nsp I and Sty I digestion steps with the Mapping 500 K Array (data not shown).

**Figure 1:**
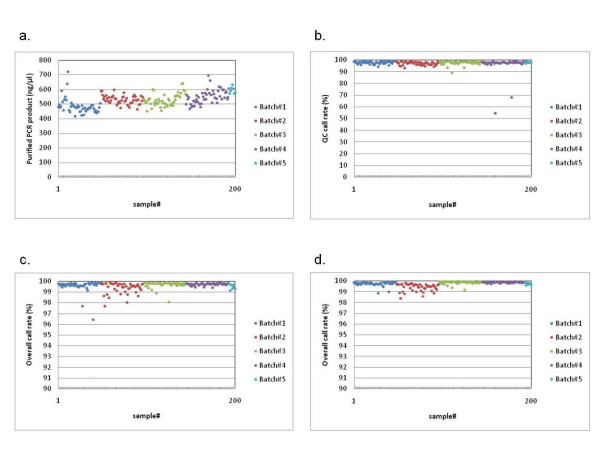
**Genotyping results of the 1^st ^set of 200 samples using the SNP Array 6.0 platform**. Colours are based on every 48 samples analyzed simultaneously as a batch. a. Concentration of purified PCR products for each sample. b. QC call rate for each sample. c. Overall call rate for each sample, as determined by the Birdseed algorithm using total 198 samples that passed the default 86% QC criteria. d. Overall call rate for each sample, as determined by the Birdseed algorithm using samples in the same batch.

**Figure 2:**
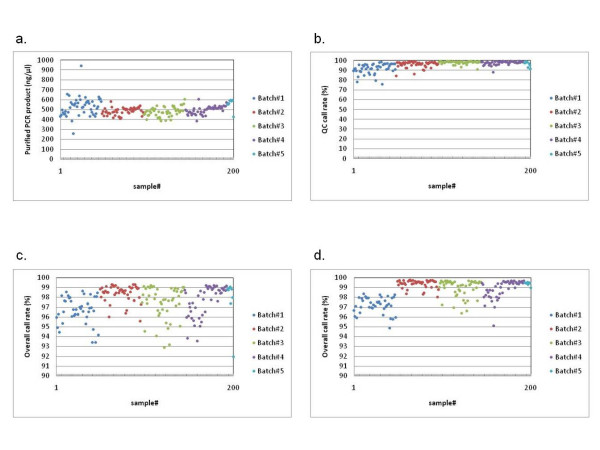
**Genotyping results of 2^nd ^set of 200 samples using the SNP Array 6.0 platform**. a. Concentration of purified PCR products for each sample. b. QC call rate for each sample. c. Overall call rate for each sample, as determined by the Birdseed algorithm using a total of 191 samples that passed the default 86% QC criteria. d. Overall call rate for each sample, as determined by the Birdseed algorithm using samples in the same batch.

The average concentration of purified PCR products for the two sets of 200 samples was 524.4 ng/μl (range 412.8 to 718.0 ng/μl) and 497.3 ng/μl (range 256.6 to 939.8 ng/μl), respectively (Figure 1a and Figure 2a). In total, 11 samples (2 samples for the 1^st ^set and 9 samples for the 2^nd ^set) showed low QC call rates below the default 86% QC criteria (Figure 1b and Figure 2b). The genotype calls of 909, 622 SNPs for each individual were determined using the Birdseed genotype calling algorithm, embedded in the Affymetrix Genotyping Console 2.0 software (Affymetrix). The 198 samples of the 1^st ^set that were over 86% QC criteria were used to assign genotypes and had an average overall call rate of 99.58%, ranging from 96.42 to 99.90% (Figure 1c). For the 2^nd ^set, 191 samples were over 86% QC criteria and the average overall call rate was 97.54%, ranging from 89.52 to 99.27% (Figure 2c). When genotype calls were determined for every 48 samples analyzed simultaneously in the same batch, the average overall call rate was improved to 99.71% (range, 98.37 to 99.94%) for the 1^st ^set, and 98.66% (range, 94.86 to 99.76%) for the 2^nd ^set (Figure 1d and Figure 2d).

### Assay criteria for experimental errors occurring on running batches

**Figure 3:**
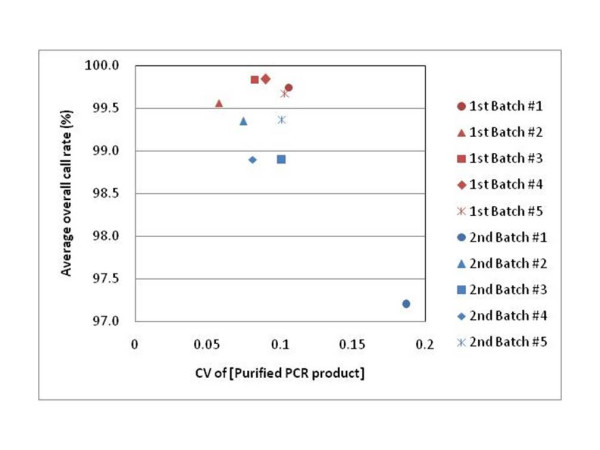
**Assay criteria for experimental errors occurring on running batches**. The CV of purified PCR product concentration is determined for each running batch. Overall call rate for each sample was determined by the Birdseed algorithm using samples in the same batch.

The SNP Array 6.0 platform has three check points prior to hybridization on GeneChip arrays in order to remove samples with experimental errors. However, some samples that pass these check points still have relatively low-quality genotyping results with lower overall call rates than 97%; 1 sample for the 1^st ^set of 200 samples and 59 samples for the 2^nd ^set of 200 samples. When genotype calls were determined for every 48 samples simultaneously analyzed in the same batch, the average overall call rate of 48 samples for batch #1 from the 2^nd ^set was 97.21%, which was almost 2% lower than other batches (Figure 2d). The concentration of purified PCR products from batch #1 drastically fluctuated among the 48 samples (Figure 2a). The CV (standard deviation/average) of the purified PCR product concentration for batch #1 was much higher than that for any other batches from the two sets of 200 samples (Figure 3). The CV of the purified PCR product concentration is a new indicator to assess experimental quality for each of the running batches, and may remove the experimental errors occurring on the running batches prior to hybridization on the GeneChip arrays.

**Figure 4:**
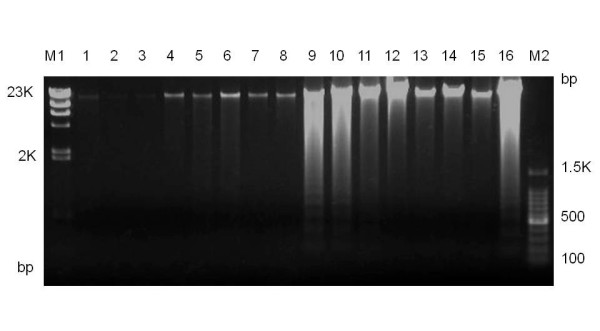
**Agarose gel electrophoresis pattern showing genomic DNA from batch #1 of the 2^nd ^set (lanes 1–8) and batch #2 of the 2^nd ^set (lanes 9–16)**. Fifty nanograms of genomic DNA for each of the sample was electrophoresed on 1.0% agarose gels. M1 and M2 indicate lambda DNA digested with Hind III and 100-bp DNA ladder marker, respectively.

For the 48 samples from batch #1 of the 2^nd ^set, the intact genomic DNA could not be detected clearly when the samples were electrophoresed on 1.0% agarose gels (Figure 4). Therefore, these genomic DNAs for batch #1 of the 2^nd ^set may have degraded due to repetitive freezing and thawing, which led to low-quality genotyping results. Preparation of the exact amount of intact genomic DNA is considered to be one of the crucial points for the SNP array 6.0 platform.

In order to assess the performance of the SNP Array 6.0 platform and the Birdseed algorithm, we mainly used genotyping data obtained from the 1^st ^set of 200 samples because the 2^nd ^set contained samples in poor condition.

### Genotype calling accuracy with "Birdseed" algorithm

**Figure 5:**
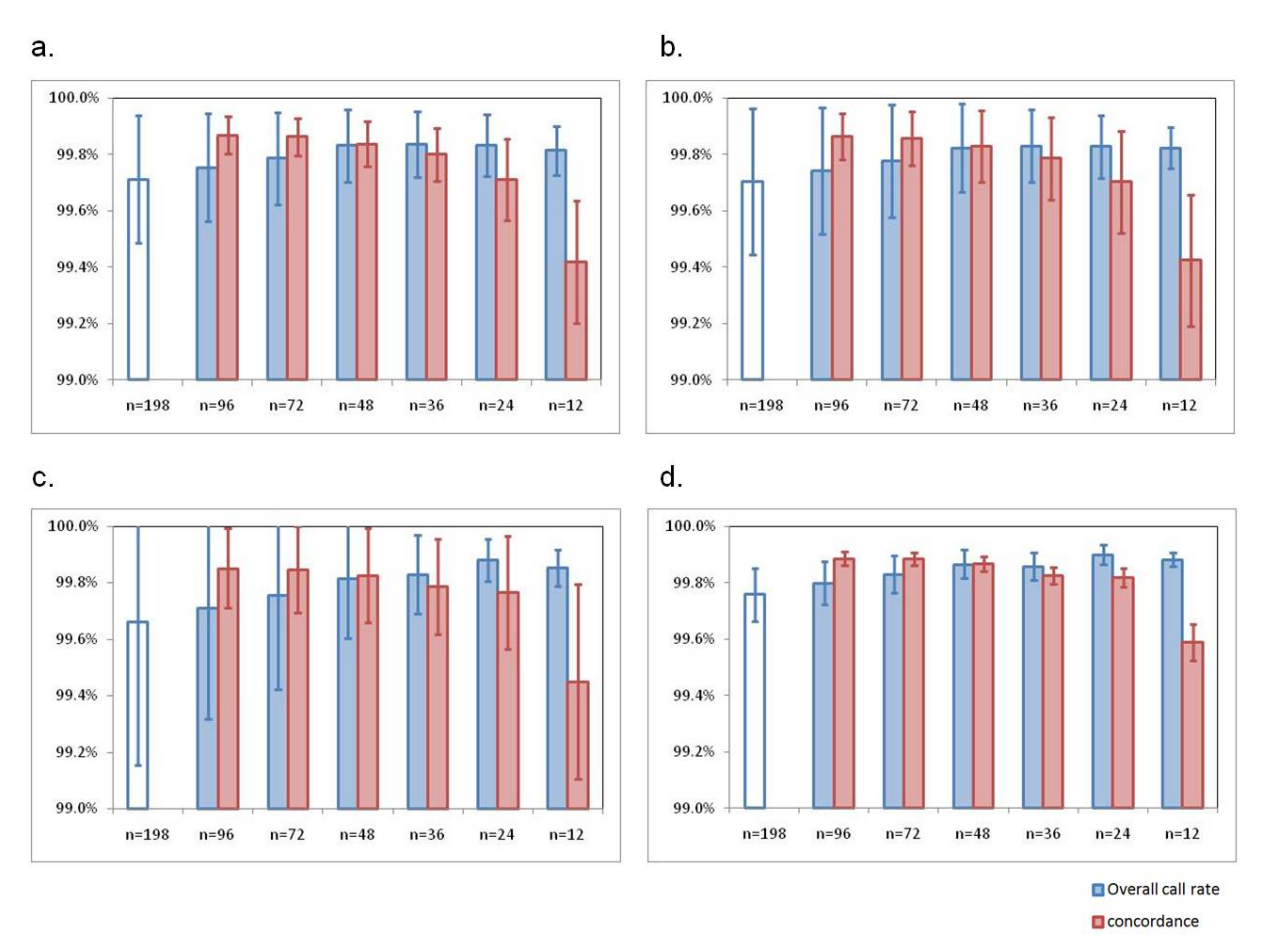
**Genotype calling accuracy with Birdseed algorithm**. a-d. Genotype calls determined using 198 samples with over 86% QC criteria were used as a reference. The average overall call rate for the 4 sets of the 12 samples were determined with 7 different sample sizes; 12 samples, 24 samples, 36 samples, 48 samples, 72 samples, 96 samples and 198 samples. The average concordance rates for the 4 sets of 12 samples were determined by comparison with the reference genotype calls. A negative correlation with a P value of 0.0053 and a positive correlation with a P value of 0.0115 were shown for overall call rate and concordance rate by fitting the power-law distribution to the data with least-squares approximation.

The genotype calling accuracy of the Birdseed algorithm was considered to be improved as the sample number for determining genotype calls increased. We determined 909,622 genotype calls for 12 samples among 198 samples with over 86% QC criteria, and used these genotype calls as a reference. We also determined the genotype calls of the same 12 samples under 6 different sample sizes, using 12 samples, 24 samples, 36 samples, 48 samples, 72 samples and 96 samples. To investigate the genotype calling accuracy of the Birdseed algorithm, we compared the genotype calls determined under 6 different sample sizes to the reference genotype calls for each of the 12 samples. We prepared 4 sets of 12 samples from a batch of 48 samples (Batch #3) and performed the genotype call comparison for each set of 12 samples. Figure 5 shows the average overall call rate and the average concordance rate for each set of the 12 samples. The average overall call rate for 4 sets of the 12 samples, which were determined with 12 samples, 24 samples, 36 samples, 48 samples, 72 samples, 96 samples and 198 samples, were 99.84%, 99.86%, 99.84%, 99.83%, 99.79%, 99.75% and 99.71%, respectively. The average concordance rate for the 4 sets of the 12 samples under 6 different sample sizes were 99.47%, 99.75%, 99.80%, 99.84%, 99.86% and 99.87%, respectively. Here, "No Calls" was excluded from the concordance calculation.

Our results showed that the average overall call rate of the 12 samples was almost constant when the genotype calls were determined with fewer than 48 samples; however, it gradually decreased as the sample number increased from 48 to 198, which showed a negative correlation with a P value of 0.0053. In contrast, the concordance rate gradually increased as the sample number increased, which showed a positive correlation with a P value of 0.0115.

### Removing low-quality samples by adjusting QC criteria

**Figure 6:**
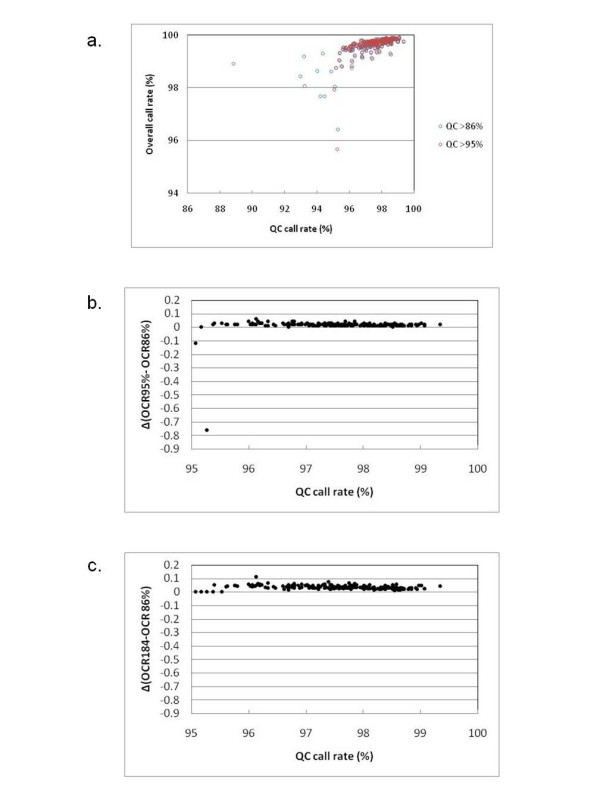
**Removal of low-quality samples by adjusting QC criteria**. Overall call rate for each sample was determined using total samples that passed the QC criteria. a. Overall call rate and QC call rate for each sample plotted with QC criteria > 86% and > 95%. b. Overall call rate (OCR) determined with 86% QC criteria compared with that determined with 95% QC criteria. c. Overall call rate (OCR) determined with 86% QC criteria compared with that determined using 184 samples.

Our results showed that the average overall call rate gradually decreased as the sample number increased, presumably due to low-quality samples included in the genotype calling with the Birdseed algorithm. Indeed, there was one sample which had an overall call rate lower than 97% among the 198 samples with over 86% QC call rate. Therefore, we applied more stringent QC criteria to remove the low-quality samples, because a linear relationship was observed between QC call rate and overall call rate (Figure 6a). When we applied 95% QC criteria, 189 samples passed the QC criteria and the average overall call rate improved from 99.58 to 99.65%. By comparing the overall call rate determined under the 95% QC criteria with that under the default criteria, 187 of 189 samples improved by an average of 0.018% in overall call rate; however, the remaining two samples showed decreased overall call rate (by 0.76% and 0.12%) (Figure 6b). These two samples were considered as outliers on the genotype calling with the Birdseed algorithm and had to be removed. We repeated the removal of samples until none had a lower overall call rate than that determined under the default criteria. A total of 184 samples had an overall call rate that improved over the one determined under the default criteria, with an average change of 0.035%. The average overall call rate for the 184 samples was 99.71%, which was 0.13% higher than the default QC criteria (Figure 6c).

### Number of SNPs available for GWAS in the Japanese population

The genotype calls of 909,622 SNPs were determined with 184 samples after sample filtering with adjusted QC criteria. However, these genotype calls still included inaccurate SNPs, which could lead to inflation of false positives, presumably due to systematically miss-called SNPs. Therefore, SNP filtering was considered to be important for a reliable and accurate set of genotype calls that avoid false association signals and false negative signals, allowing rapid identification of disease susceptibility genetic factors. We reported that the poorly behaving SNPs were effectively eliminated with the SNP filtering parameters; MAF > 5% or 1%, HWE p-value > 0.001 and SNP call rate > 95% [14]. Here, SNP call rate was defined for each SNP as the number of successfully genotyped samples divided by the number of total samples genotyped.

Among a total of 909,622 SNPs genotyped using 184 samples, 590,248 SNPs passed the three SNP filtering criteria with MAF > 5%, HWE p-value > 0.001 and SNP call rate > 95%, while 661,559 SNPs passed with MAF > 1%, HWE p-value > 0.001, and SNP call rate > 95%. A total of 180,859 SNPs were observed to be monomorphic in the Japanese population.

## Discussion

The emerging SNP typing technologies have enabled genome-wide association studies to be conducted with hundreds of thousands of genotyped SNPs. According to Affymetrix, the SNP Array 6.0 platform can genotype over 900 K SNP markers across the human genome with an overall call rate of at least 97%, over 99.7% concordant with the HapMap genotypes, and the Mendelian inheritance consistency for 10 Trios of greater than 99.9% when performing analysis under the default 86% QC criteria. To evaluate the SNP 6.0 Array platform and the Birdseed genotype calling algorithm, we genotyped two sets of 200 non-HapMap Japanese samples using the SNP Array 6.0 platform.

When we applied the default 86% QC criteria, 2 samples out of the 1^st ^set of 200 samples were excluded and the average overall call rate was 99.58%. There was one sample with an overall call rate of lower than 97% among the 198 samples. Here, we found a linear relationship between QC call rate and overall call rate. Therefore, we applied stringent QC criteria of over 95% in order to remove the low-quality samples and found that the average overall call rate for 189 samples passing the stringent QC criteria improved to 99.65%. Among the 189 samples, 187 samples had higher overall call rates than those determined under the default QC criteria; however, the remaining two samples showed lower overall call rates (by 0.76% and 0.12%). When we repeated the removal of samples until none had a lower overall call rate than the one determined under the default criteria, none of the remaining 184 samples with an overall call rate lower than 97%. The average overall call rate of 184 samples was thus improved to 99.71%. The decay of average overall call rate may be caused by some samples that pass the QC criteria, but still have a low overall call rate. We can thus improve overall call rate by removing these samples and adjusting the QC criteria.

One of the crucial points for the SNP array 6.0 platform is to prepare the exact amount of intact genomic DNA. A 10-fold excess amount of genomic DNA decreased the overall call rate of each sample to by about 80% and another study revealed that samples with less than 50 ng/μl genomic DNA show low overall call rates [15]. Therefore, we checked the concentration and condition of genomic DNA with the NanoDrop quatitation and agarose gel electrophoresis. The SNP array 6.0 platform has three check points to assess experimental errors prior to hybridization on GeneChip arrays. Here, we found that the CV of the purified PCR product concentration was another critical indicator prior to hybridization in assessing the performance of each running batches. We suggest that samples with a CV value over 0.15 are excluded from the remainder of the assay.

The genotype calling accuracy of the Birdseed algorithm was assessed by comparing the 909,622 genotype calls of 12 samples from among198 samples with over 86% QC criteria, to those of 12 samples determined with six different sample sizes; 12 samples, 24 samples, 36 samples, 48 samples, 72 samples and 96 samples. The concordance rate gradually increased as the number of samples increased. The average concordance rate was almost constant over 99.8%, when the genotype calls were determined with over 48 samples using the Birdseed algorithm. However, the average overall call rate of the 12 samples gradually decreased as the sample number increased from 48 to 198. We could explain the reasons why the overall call rate decreases, and why the concordance rate increases for these 12 samples in a grouping of samples greater than 48 by means of characteristic properties of the Birdseed algorithm and minor allele frequency of each SNP. When the sample number was smaller than 48, all of three clusters designating AA, AB and BB genotypes were rarely observed for the SNPs with low MAF. In such cases, the Birdseed algorithm would determine the genotype as a single cluster, however, would ambiguously genotype as AA, BB and AB (tend to miss-genotype). Therefore, high call rate and low concordance were observed with the sample number smaller than 48. In contrast, when the sample number was greater than 48, two or three clusters would be observed for many SNPs. For these SNPs, the Birdseed algorithm could determine the outlying samples from each cluster as "No Calls", leading to low call rate and high concordance.

We can accurately determine the genotype calls with high overall call rates by determining the genotype calls with more than 48 samples, after removing low-quality samples by adjusting the QC criteria. Our results showed that the SNP Array 6.0 platform reached the expected level reported by the manufacturer, with an average overall call rate of over 99.5% and an average concordance rate of over 99.8%. However, about 20% of a total of 909,622 SNPs were found to be monomorphic in the Japanese population, which is due to SNP selection methods. The SNPs assayed on the SNP Array 6.0 platform were mainly selected as observed with high MAF in the Caucasian population. Among a total of 909,622 SNPs genotyped using the SNP Array 6.0 platform with 184 Japanese samples, 590,248 SNPs passed three SNP filtering criteria; MAF > 5%, HWE p-value > 0.001 and SNP call rate > 95%. Although the exact number of SNPs within the human genome remains under discussion, it has been reported that the genome coverage of the JPT + CHB population in the Phase II HapMap data was 66% using the Mapping 500 K Array set [10]. The genome coverage of the SNP array 6.0 platform was estimated using the same calculation and was revealed to be 75% with the 590,248 SNPs in the Japanese population.

## Conclusion

The current Affymetrix SNP Array 6.0 platform enables the genotyping of over 900 K SNPs with high overall call rate (over 99.5%) and high concordance rate (over 99.8%). The number of SNPs available for GWAS in the Japanese population was revealed to be over 660 K SNPs, all of which passed the three SNP filtering criteria; MAF > 1%, HWE p-value > 0.001 and SNP call rate > 95%. GWAS using the SNP Array 6.0 platform has considerable potential in identifying candidate susceptibility or resistance genetic loci for multifactorial diseases in the Japanese population, as well as in other populations.

Finally, the genotyping data of 400 Japanese samples using the SNP array 6.0 platform will be deposited in a public database to share with the research community [16].

## Methods

### Study sample

Blood samples were obtained from two sets of 200 Japanese individuals in two institutes. Genomic DNA was extracted from peripheral blood leukocytes using the QIAamp Blood Mini Kit (Qiagen) according to the manufacturer's instructions. All genomic DNA was resuspended with Reduced EDTA TE Buffer (TEKnova) at 50 ng/μl. This study was approved by the Research Ethics Committee of the Faculty of Medicine, The University of Tokyo and Tokai University. Informed consent was obtained from all participants.

### Genotyping 400 Japanese samples with SNP Array 6.0 platform

The concentration of genomic DNA for all individuals was measured using a spectrophotometer (NanoDrop ND-1000, NanoDrop Technologies). For the 1^st ^set of 200 samples, five samples had low genomic DNA concentrations with an average of 41.1 ng/μl ranging from 38.2 to 44.5 ng/μl, and the remaining 195 samples had an average of 54.8 ng/μl, ranging from 45.0 to 57.8 ng/μl. For the 2^nd ^set of 200 samples, one sample had 39.1 ng/μl and the remaining 199 samples had an average of 52.7 ng/μl, ranging from 45.0 to 55.9 ng/μl. For each individual assayed, 250 ng of genomic DNA was digested with Sty I and Nsp I (New England BioLabs) by adding 6 μl for the 6 samples with low concentration (five samples for 1^st ^set and one sample for 2^nd ^set) and 5 μl for the remaining samples. For two sets of 200 samples, every 48 samples were simultaneously processed in a single 96-well plate. After the reaction with restriction enzymes, we followed the manufacturer's instructions for the Affymetrix Genome-wide Human SNP array 6.0. The concentration of PCR products after purification with magnetic beads (Agencourt Magnetic Beads, Beckman) was measured using a spectrophotometer (NanoDrop ND-1000). Purified PCR products were diluted 10-fold with TE buffer (pH 8.0) (WAKO) in order to have a suitable concentration for the spectrophotometer to measure. The genotype calls of each individual were determined by the Birdseed version 1 genotype calling algorithm, embedded in the software Affymetrix Genotyping Console 2.0 (Affymetrix). The number of samples used to determine the genotype calls varied depending on the examination.
